# Computer-Based Cognitive Programs for Improvement of Memory, Processing Speed and Executive Function during Age-Related Cognitive Decline: A Meta-Analysis

**DOI:** 10.1371/journal.pone.0130831

**Published:** 2015-06-22

**Authors:** Yan-kun Shao, Jing Mang, Pei-lan Li, Jie Wang, Ting Deng, Zhong-xin Xu

**Affiliations:** 1 Department of Neurology, China-Japan Union Hospital, Jilin University, Changchun, China; 2 Department of Emergency, Beijing Bo Ai Hospital & China Rehabilitation Research Center, Capital Medical University, Beijing, China; University Of São Paulo, BRAZIL

## Abstract

**Background:**

Several studies have assessed the effects of computer-based cognitive programs (CCP) in the management of age-related cognitive decline, but the role of CCP remains controversial. Therefore, this systematic review evaluated the evidence on the efficacy of CCP for age-related cognitive decline in healthy older adults.

**Methods:**

Six electronic databases (through October 2014) were searched. The risk of bias was assessed using the Cochrane Collaboration tool. The standardized mean difference (SMD) and 95% confidence intervals (CI) of a random-effects model were calculated. The heterogeneity was assessed using the Cochran Q statistic and quantified with the *I^2^* index.

**Results:**

Twelve studies were included in the current review and were considered as moderate to high methodological quality. The aggregated results indicate that CCP improves memory performance (SMD, 0.31; 95% CI 0.16 to 0.45; p < 0.0001) and processing speed (SMD, 0.50; 95% CI 0.14 to 0.87; p = 0.007) but not executive function (SMD, -0.12; 95% CI -0.33 to 0.09; p = 0.27). Furthermore, there were long-term gains in memory performance (SMD, 0.59; 95% CI 0.13 to 1.05; p = 0.01).

**Conclusion:**

CCP may be a valid complementary and alternative therapy for age-related cognitive decline, especially for memory performance and processing speed. However, more studies with longer follow-ups are warranted to confirm the current findings.

## Introduction

Age-related cognitive decline, as a non-pathological loss in cognitive function, is common in older adults [1–5] that hampers the basic activities of daily life, resulting in a loss of personal autonomy [6–8]. The loss of independence is a key feature of dementia and is a source of both physical and psychological stress for older adults [9]. Therefore, there is a growing demand for effective, low-cost solutions to improve or delay age-related cognitive decline.

Cognitive training programs have been offered to prevent or minimize age-related cognitive decline and usually consist of guided practice of specific cognitive tasks or cognitive stimulation programs aimed at improving general cognition [10]. Previous studies indicated that traditional cognitive training programs could enhance cognitive functions, such as memory performance, processing speed and executive function in healthy older adults [10–12].

With the development of new technologies, computer-based cognitive programs (CCP), which promise a series of advantages over traditional cognitive training, have been rapidly disseminated to older adults. CCP can set the initial level of task difficulty according to the participants’ baseline competency and can be gradually adjusted as performance improves, which keeps the activity engaging and fun. Furthermore, CCP enable the standardization of intervention [13]. In the last two decades, CCP have been well developed, and some clinical trials reported CCP delayed cognitive decline and improved cognitive function in healthy older adults [14–18]. However, the role of CCP in the management of age-related cognitive decline remains controversial. A previous review also reported that computerized cognitive training was an effective, less labor intensive alternative for treating cognitive impairment [19], but it was only a descriptive review without a quantitative analysis.

Therefore, this review evaluated the evidence on the efficacy of CCP for age-related cognitive decline in healthy older adults. The meta-analyses in this review focused on memory performance, processing speed and executive function. Based on our findings, we offer recommendations for future research.

## Materials and Methods

### Search strategy

Electronic databases (to October 2014) were searched: PubMed, EMBASE, Cochrane Library, China Knowledge Resource Integrated Database, Wan Fang Data and Weipu Database for Chinese Technical Periodicals. The following keywords were used in various combinations: older adult(s), elderly, aging, aged, cognition, cognitive ability, cognitive function, computer(s), computerized training, video and game(s). To identify unpublished studies, trial registrations (WHO International Clinical Trials Registry Platform) and dissertations (Chinese Dissertation Full-text Database and ProQuest Dissertations) were searched. The reference lists of relevant articles were reviewed for additional trials, and we contacted experts in the field.

### Study selection

The studies that met the following criteria were included: (1) study design: randomized controlled trials (RCTs); (2) the participant: healthy older adults (> 60 years old); (3) the intervention: CCP including modified cognitive training tasks, video games or neuropsychological software designed to improve cognitive functions, such as memory, processing speed, executive function, etc.; (4) the outcomes: memory, executive function, processing speed; and (5) the publication was available in either English or Chinese. Studies were excluded based on the following criteria: (1) participants were diagnosed with Alzheimer’s disease or other forms of senile dementia; (2) the outcomes did not include any cognitive functions; and (3) participants were mixed with younger and older adults. After the exclusion of duplicates, two authors independently identified and selected the studies. Disagreements were resolved by discussion among the authors.

### Data extraction

Two authors independently extracted data from the studies. The following contents were extracted: general information (study source, sample size and age), details of the intervention (type, duration and dose), main outcomes and the follow-up duration. Disagreements were resolved by discussion.

### Risk of bias

The risk of bias was assessed independently by two authors using the Cochrane Collaboration tool [20,21]. The following domains were assessed: 1) random sequence generation, 2) allocation concealment, 3) blinding of participants and personnel, 4) blinding of outcome assessments, 5) incomplete outcome data and 6) selective reporting. Every domain was classified as “low risk of bias”, “high risk of bias” or “unclear risk of bias”. The necessary information was supplemented by contacting the corresponding author. There were no disagreements on the risk of bias assessments among the authors.

### Data synthesis and analysis

The meta-analyses were conducted using the Cochrane Collaboration software (Review Manager Version 5.2). For continuous data, the standardized mean difference (SMD) and 95% confidence intervals (CI) were calculated in the meta-analysis. In order to get expected heterogeneity, random-effects model was employed in summary estimates of the treatment effect. The heterogeneity was assessed by the Cochran Q statistic (considered significant when the P value was less than 0.10) and was quantified using the *I* ^2^ index (considered high when *I* ^2^ was greater than 75%). Similar intervention groups were combined using the formula of the Cochrane handbook for a single pair-wise comparison. Subgroup analyses were conducted based on outcome measures. Potential publication bias was assessed by visually inspecting of the Begg funnel plots.

## Results

### Literature search

**Fig 1:**
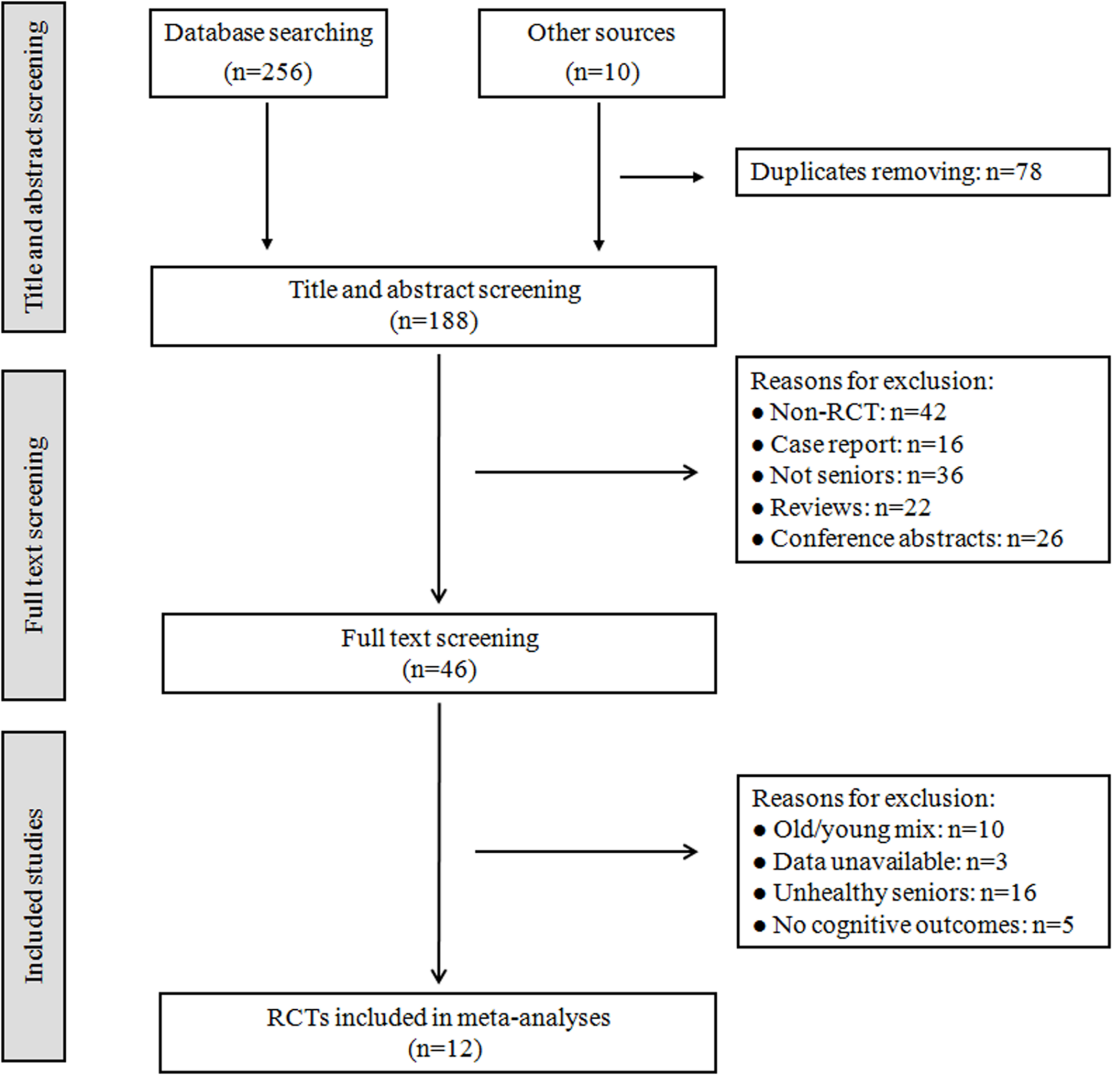
Flow diagram of study selection. RCTs: randomized controlled trials.

The detailed search process is illustrated in Fig 1. A total of 188 records were retrieved after removing duplicates. In total, 46 full-text publications were identified after screening the titles and abstracts. Based on the inclusion criteria, 12 studies were included in the review [14–18,22–28]. During the full-text screening, 34 studies were excluded due to unavailable data [29–31] (n = 3), no cognitive measures (n = 5), old/young mix (n = 10) and unhealthy seniors (n = 16). During conducting this review, the related experts were contacted, but there were not more information such as unpublished data, ongoing research, etc.

### Study characteristics

The characteristics of all the included studies are summarized in Table 1. In total, 12 RCTs with 2008 participants were included in our review [14–18,22–28]. All of the studies were published in English. The study duration ranged from 4 weeks to 24 weeks. The training programs were practiced 10 to 75 sessions (mean ± SD: 32.67 ± 17.84) ranging from 15 to 120 minutes each session (mean ± SD: 55.83 ± 29.19). The follow-up duration ranged between 12 and 48 weeks. The control interventions included no contact control [14,16], education [15,16,22–25], no training [17], physical exercise [18,23], Tetris [26], computer-based daily online news [27] and waiting list [28].

**Table 1:** Characteristics of included studies.

First author, year, country	Sample size	Age: mean or range (year)	Dose	Follow-up (week)	Main outcome Assessments	Experimental group Intervention (Game name)	Control group intervention
Edwards, 2002, US[14]	97	74	60 min, 10 sessions, 6 wk	—	Digit span, Stroop test, Trial making test A and B, Digit symbol subset	Computerized speed of processing training	No contact control
Edwards, 2005, US[15]	181	76	60 min, 10 sessions, 5 wk	—	Digit span, Stroop test, Trial making test A and B, Digit symbol subset	Computerized speed of processing training	Computerized education
Mahncke, 2006, US[16]	182	71	60 min, 40–50 sessions, 8–10 wk	12	Digit span, Processing speed	Brain plasticity–based computerized cognitive Training	1) Video-education; 2) No-contact control
Basak, 2008, US[17]	40	69	120 min, 21–24 sessions, 7–8 wk	—	Visual short-term memory, Operation span	Real-time strategy video game	No training
Buschkuehl, 2008, Switzerland[18]	39	80	32 min, 24 sessions, 12 wk	48	Digit span, Verbal free recall, Viaual free recall	Computerized cognitive training(Working memory and reaction time tasks)	Physical exercise
Smith, 2009, US[22]	487	75	60 min, 40 sessions, 8 wk	—	Digit span, Letter-number sequencing, Immediate recall, Delayed recall, Overall memory, Processing speed	Brain plasticity–based computerized cognitive training	Video-education
Klusmann 2010, Germany[23]	259	70–93	90 min, 75 sessions, 24 wk	—	Immediate recall, Delayed recall, Stroop test, Trail making tests A/B	Computerized cognitive training	1) Physical exercise; 2) Education
Stern, 2011, US[24]	60	66	60 min, 36 sessions, 12 wk	12	Letter-number sequencing, Digit symbol subset, Trial making test A and B	1) Emphasis change game training (Space Fortress)2) Standard game training (Space Fortress)	Education
Zelinski, 2011, US[25]	487	75	60 min, 40 sessions, 8–10 wk	12	Letter-number sequencing, Digit span, Immediate recall, Delayed recall, Overall memory, Processing speed	Brain plasticity–based computerized cognitive Training	Video-education
Nouchi, 2012, Japan[26]	32	69	15 min, 20 sessions, 4 wk	—	Frontal assessment battery at bedside, Trial making test B, Digit symbol coding and symbol search	Brain game training(Brain Age)	Tetris
Bozoki, 2013, US[27]	60	68	30 min, 30 sessions, 6 wk	—	Delayed recall, MON-monitoring, IDN-identification	Computerized game training (My Better Mind)	Computer-based daily online news
Miller, 2013, US[28]	84	82	20–25 min, 40 sessions, 8 wk	16	WMS-III	Computerized cognitive training (Dakim’s Brain Fitness)	Waiting list

Abbreviations: wk = week; MON = Monitoring (simple reaction time); IDN = Identification (visual-motor reaction time); WMS-III = Wechsler memory scale III.

### Risk of bias

**Fig 2:**
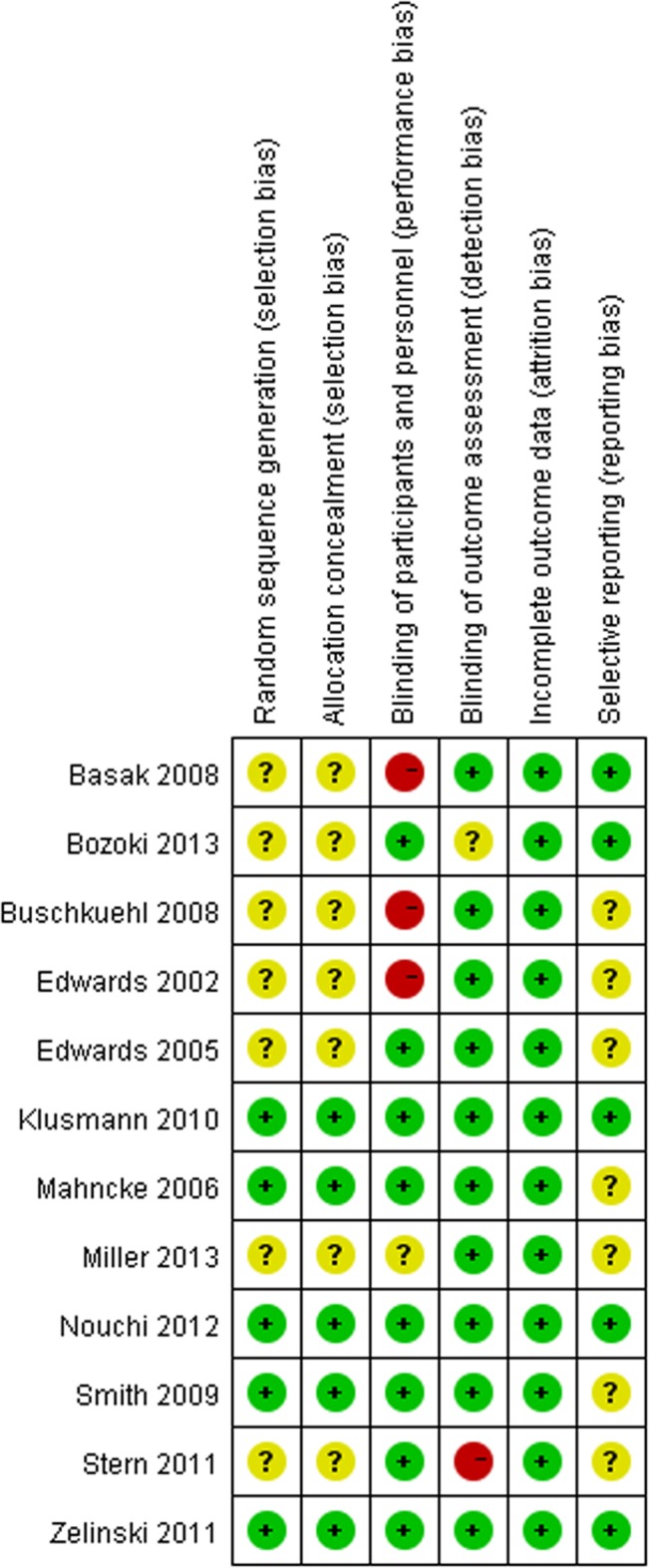
Risk of bias. Red (-): high risk of bias; Yellow (?): unclear risk of bias; Green (+): low risk of bias.

The risk of bias in the individual studies is presented in Fig 2. In total, 5 trials [16,22,23,25,26] reported adequate methods of random sequence generation and allocation concealment, and the other studies were unclear [14,15,17,18,24,27,28]. Most trials (83%) [14–18,22,23,25,26,28] used independent outcome assessors who were unaware of the group assignment. Although 3 studies were at high risk for bias [14,17,18], 8 trials (67%) [15,16,22–27] blinded the participants. For incomplete outcome data, all trials [14–18,22–28] were at low risk. 5 trials [17,23,25–27] were at low risk for selective reporting. In assessing the bias, necessary information was supplemented or clarified by contacting study-authors, especially for selective reporting [23,25,26].

### Quantitative data synthesis

#### Memory

**Fig 3:**
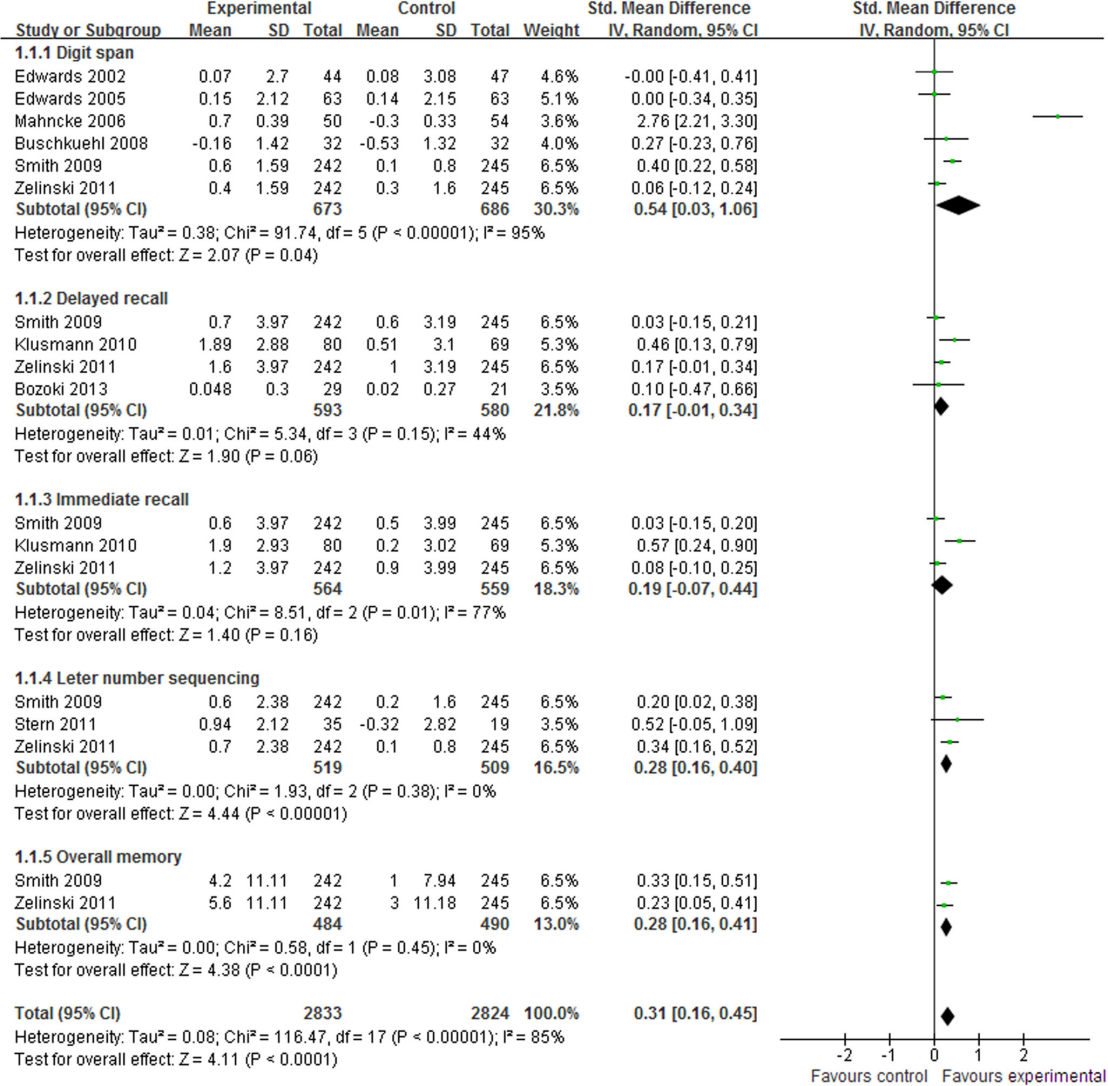
Forest plot of the effect of computer-based cognitive programs in memory performance.

Memory is the ability to retain, store and recall information. Age-related memory loss is a universal condition for older adults. In total, 10 studies [14–18,22–25,27] assessed the effect of CCP in memory performance. The aggregated results indicated that CCP improved memory performance (SMD, 0.31; 95% CI 0.16 to 0.45; p < 0.0001, Fig 3) compared to the control interventions. In the subgroup meta-analyses, CCP demonstrated significant effects in digit span (SMD, 0.54; 95% CI 0.03 to 1.06; p = 0.04, Fig 3) [14–16,18,22,25], letter-number sequencing (SMD, 0.28; 95% CI 0.16 to 0.40; p < 0.00001, Fig 3) [22,24,25] and over memory (SMD, 0.28; 95% CI 0.16 to 0.41; p < 0.0001, Fig 3) [22,25]. CCP were also associated with small but not significant improvements in delayed recall (SMD, 0.17; 95% CI -0.01 to 0.34; p = 0.06, Fig 3) [22,23,25,27] or immediate recall (SMD, 0.19; 95% CI -0.07 to 0.44; p = 0.18, Fig 3) [22,23,25].

#### Processing speed

**Fig 4:**
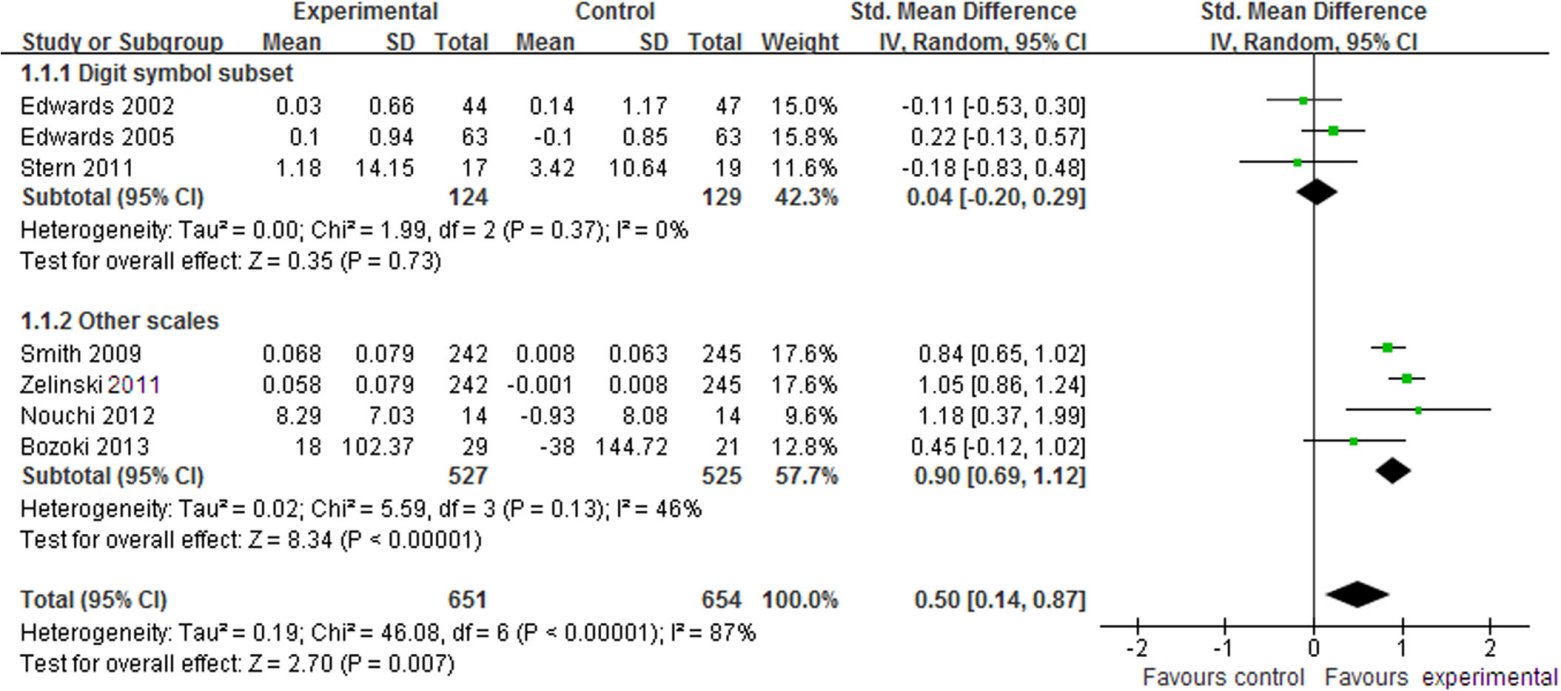
Forest plot of the effect of computer-based cognitive programs in processing speed.

Processing speed is the ability to quickly process information. In total, 8 studies [14–16,22,24–27] assessed the effect of CCP on processing speed. There was no significant difference between CCP and control interventions in digit symbol subset (SMD, 0.04; 95% CI -0.20 to 0.29; p = 0.73, Fig 4) [14,15,24] but CCP demonstrated better effects in other assessment scales (SMD, 0.90; 95% CI 0.69 to 1.12; p < 0.00001, Fig 4) [22,25–27]. Furthermore, the aggregated results indicated that CCP were associated with significant improvements in processing speed (SMD, 0.50; 95% CI 0.14 to 0.87; p = 0.007, Fig 4).

#### Executive function

**Fig 5:**
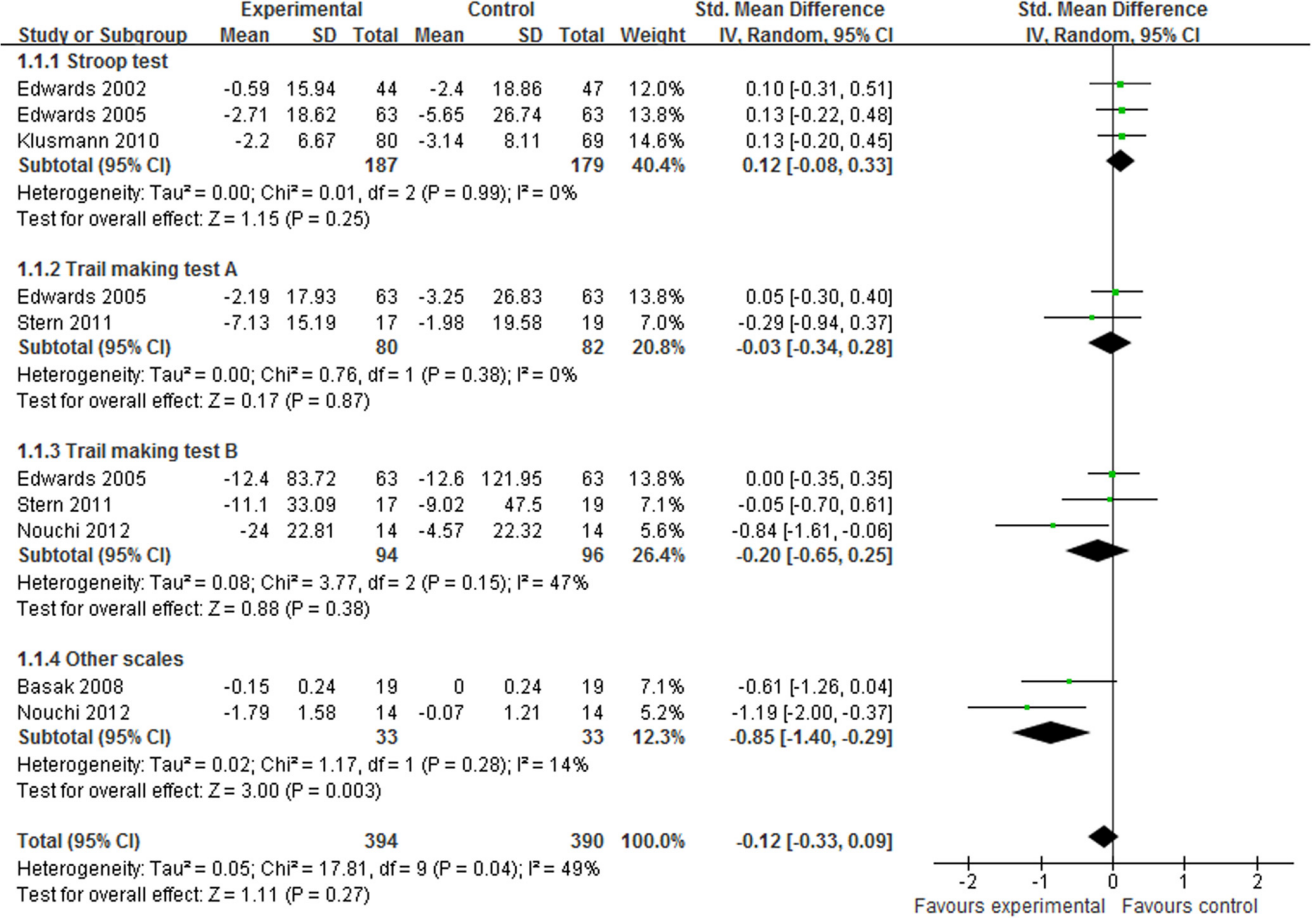
Forest plot of the effect of computer-based cognitive programs in executive function.

Executive function consists of a broad spectrum of abilities, including cognitive flexibility, planning, and abstract thinking skills. Older adults usually have poorer executive function due to cognitive decline [32]. In total, 6 studies [14,15,17,23,24,26] assessed the effect of CCP on executive function. The aggregated results indicated that there was no significant difference between CCP and control interventions on executive function (SMD, -0.12; 95% CI -0.33 to 0.09; p = 0.27, Fig 5). In the subgroup meta-analyses, the results did not support the effect of CCP in the Stroop test (SMD, 0.12; 95% CI -0.08 to 0.33; p = 0.25, Fig 5) [14,15,23], trail marking test A (SMD, -0.03; 95% CI -0.34 to 0.28; p = 0.87, Fig 5) [15,24] or trail marking test B (SMD, -0.20; 95% CI -0.65 to 0.25; p = 0.38, Fig 5) [15,24,26].

#### The long-term effect

**Fig 6:**
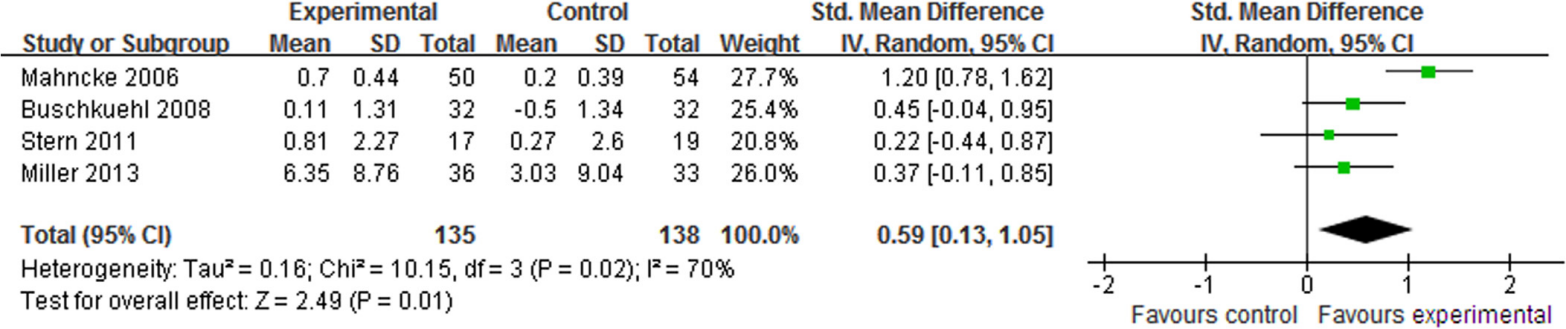
Forest plot of the long-term effect of computer-based cognitive programs in memory performance.

In total, 5 studies [16,18,24,25,28] assessed the long-term effect of CCP for age-related cognitive decline. Some studies reported that there was no significant difference between CCP and control interventions, but the aggregated results indicate that CCP had better long-term effects on memory performance (SMD, 0.59; 95% CI 0.13 to 1.05; p = 0.01, Fig 6) [16,18,24,28]. Significant differences in the improvements from baseline to the 3-month follow-up were also reported between brain plasticity-based computerized cognitive training and video education in processing speed and attention [25].

#### Publication bias

**Fig 7:**
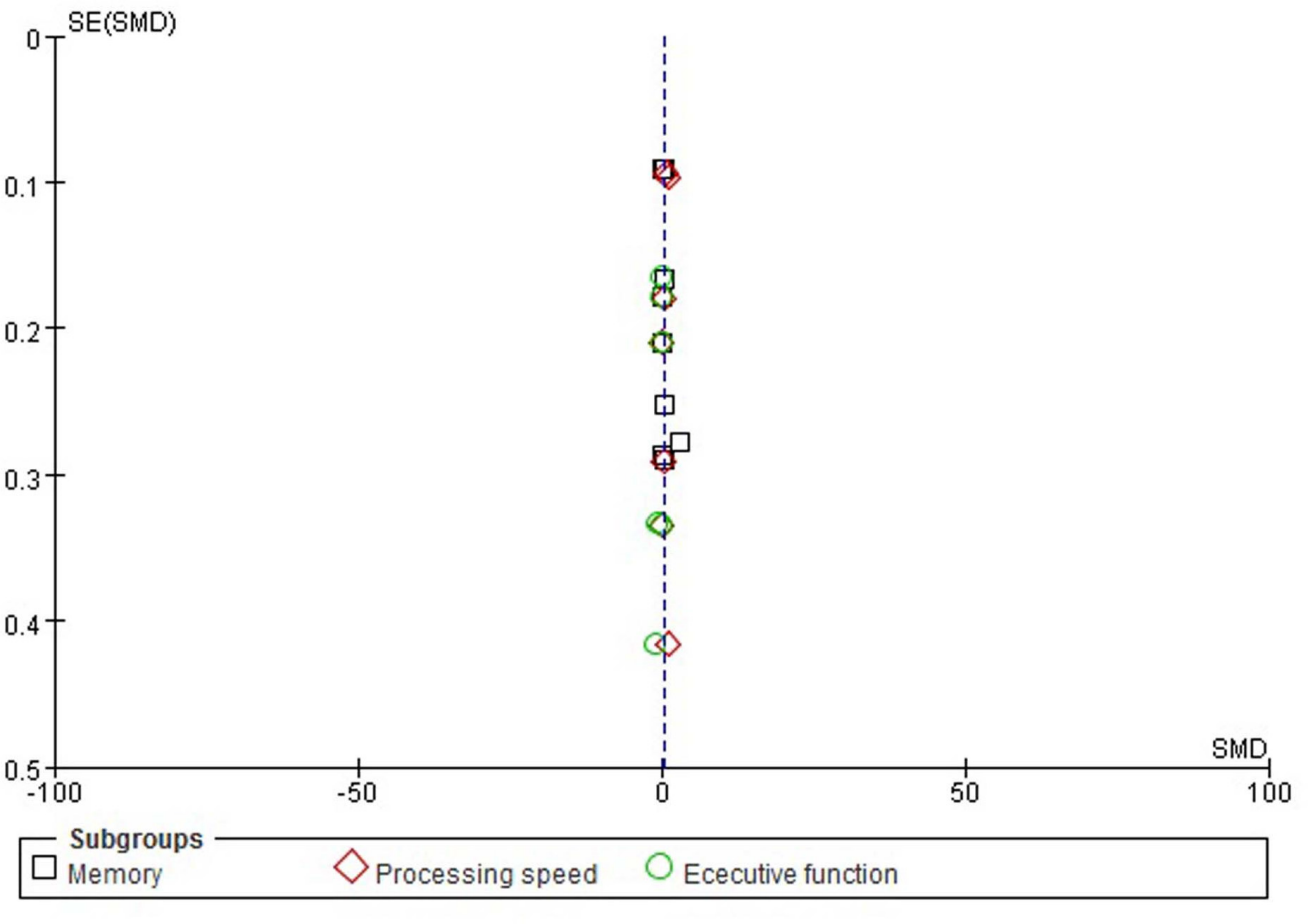
Funnel plot for memory, processing speed, and executive function.

The funnel plots for memory, processing speed, and executive function were performed (Fig 7), but it is difficult to interpret the result of publication bias due to such a small subset of studies.

## Discussion

The current review evaluated the evidence on the effects of CCP in age-related cognitive decline in healthy older adults. In total, 12 moderate to high quality studies were included in our review. The aggregated results indicated that CCP improved memory performance and processing speed. There was also a long-term enhancement in memory performance.

Our positive results concur with previous reviews [10,19]. The last review conducted by Kueider and his colleagues concluded that computerized training was an effective, less labor intensive alternative compared with traditional, paper-and-pencil cognitive training [19]. However, it was only a qualitative review with both RCTs and non-RCTs published from 1986 to 2011. Any strictly qualitative approach may be problematic because it is more subjective than a meta-analysis. In our review, 12 moderate to high quality RCTs were included [14–18,22–28]. Detailed subgroup meta-analyses were conducted based on the different outcome measures. The aggregated results indicated that CCP improved memory performance and processing speed. Furthermore, there was a long-term enhancement in memory performance. Consequently, the current review provides stronger evidence of CCP beneficial effects on age-related cognitive decline.

Compared with traditional cognitive training, CCP have several advantages. It is a more convenient, cost-effective training strategy for older adults with cognitive decline. With the development of modern technology, ownership of personal computers has grown sharply, and older adults have access to CCP. Computerized training also requires less face-to-face training. Thus, trainer costs can be significantly reduced. Furthermore, CCP offer self-paced, individualized training, which sets the initial level of task difficulty according to the baseline competency of the participants and gradually adjusts it as they improve performance (keeping the activity engaging and fun). These advantages promote the application and popularization of CCP.

The CCP-related improvements in cognitive function may be associated with neuroplasticity. Neuroplasticity is a long-term physiological process of structural and functional self-repair in the brain [33]. Enhancing plasticity in the older adult brain may slow the progression of cognitive decline and improve specific cognitive functions, such as memory, attention, language and executive function [16,22,25]. Computer-based cognitive training usually consists of programmed tasks, which may adjust the complexity of the training based on the user’s performance. This programmed stimulation is beneficial for neuroplasticity in the brain [16,22]. Brain plasticity-based computerized cognitive training indicated favorable gains in memory performance, processing speed and attention in older adults [16,22,25]. Brain imaging findings support that neuroplasticity training may increase the activation of damaged brain regions, which may improve cognitive function and facilitate recovery from neurological diseases [34]. Previous studies also demonstrated that cognitive training might benefit plasticity by improving sensory systems in the cerebral cortex [35,36].

There were some potential limitations in our review. 1) Some aggregated results may have been influenced by high heterogeneity, especially for immediate recall and digit span. The main reason was that different computerized tasks were used in eligible studies. These tasks contained not only simple arithmetic calculations, but also complex logic puzzle games designed to engage spatial executive processing and non-verbal reasoning. Although tasks may continuously adjust difficulty based on user performance, their complexity was obviously different. Furthermore, dosage of computerized interventions may affect the aggregated results. It ranged from 4 weeks to 24 weeks in eligible studies. Longer treatment period may result in better improvements. In addition, the explanation could be age differences, but there are not obvious age differences among eligible studies. 2) Only studies published after 2000 were included in our review because the criteria for mild cognitive impairment were not defined until 1999 [37]. Some patients with mild cognitive impairment may be included in earlier studies. 3) There was a degree of uncertainty in identifying the relevant studies due to limited retrieval resources, language barriers, and publication bias.

## Conclusions

In this review, there was positive evidence of CCP benefits for age-related cognitive decline in healthy older adults. CCP should be recommended as a complementary and alternative therapy for age-related cognitive decline, especially in memory performance and processing speed. However, more well-designed RCTs with longer follow-ups are warranted to confirm the current findings.

## Supporting Information

S1 PRISMA Checklist PRISMA Checklist. (DOC)
